# Hemophagocytic Lymphohistiocytosis in Intensive Care Unit: A 71-Case Strobe-Compliant Retrospective Study

**DOI:** 10.1097/MD.0000000000002318

**Published:** 2015-12-28

**Authors:** Thomas Barba, Delphine Maucort-Boulch, Jean Iwaz, Julien Bohé, Jacques Ninet, Arnaud Hot, Jean-Christophe Lega, Claude Guérin, Laurent Argaud, Christiane Broussolle, Yvan Jamilloux, Jean-Christophe Richard, Pascal Sève

**Affiliations:** From the Hospices Civils de Lyon, Department of Internal Medicine, Croix-Rousse University Hospital, Lyon; Université Lyon I, Villeurbanne (TB, CB, YJ, PS); Hospices Civils de Lyon, Service de Biostatistique, Lyon; CNRS UMR 5558, Equipe Biostatistique-Santé, Pierre-Bénite; Université Lyon I, Villeurbanne (DM-B, JI); Hospices Civils de Lyon, Intensive Care Unit, Centre Hospitalier Lyon Sud, Pierre-Bénite; Université Lyon I, Villeurbanne (JB); Hospices Civils de Lyon, Department of Internal Medicine, Edouard Herriot University Hospital, Lyon; Université Lyon I (JN, AH); Hospices Civils de Lyon, Department of Internal Medicine, Centre Hospitalier Lyon Sud, Pierre-Bénite; Université Lyon I (JCL); Hospices Civils de Lyon, Intensive Care Unit, Croix-Rousse University Hospital, Lyon; Université Lyon I, Villeurbanne (CG, JCR); and Hospices Civils de Lyon, Intensive Care Unit, Edouard Herriot University Hospital, Lyon; Université Lyon I, Villeurbanne, France (LA).

## Abstract

Hemophagocytic lymphohistiocytosis (HLH) is a critical condition that may lead to organ failure and early death. The aim of this retrospective observational study was to describe a cohort of HLH patients admitted to intensive care unit (ICU) and investigate the risk factors of early death.

A positive HLH diagnosis was defined by an HScore ≥169. Univariate and multivariate analyses were carried out to investigate hospital and 28-day mortality risk factors. Between January 2002 and July 2014, 71 HLH cases were seen at our institution.

The overall 28-day mortality (start at ICU admission) and hospital mortality were 38% and 68%, respectively. The factors associated with increased 28-day mortality were the sequential organ failure assessment score at ICU admission (*P* < .001) and advance in age (*P* = 0.03). The factors associated with increased hospital mortality were a high sequential organ failure assessment score at ICU admission (*P* < 0.01), advance in age (*P* = 0.04), and the presence of lymphoma-related HLH or HLH of unknown origin (*P* < 0.01).

Organ failure overtops the classical early-death risk factors in adult ICU-admitted HLH patients. This failure and the subsequent early death may be prevented by timely specific cytotoxic therapies and the control of the underlying disease.

## INTRODUCTION

Hemophagocytic lymphohistiocytosis (HLH) is a life-threatening condition characterized by a hyperinflammatory state mediated by impaired natural-killer (NK) and cytotoxic-T-cell functions.^1^ The main clinical features of HLH include high fever, hepatosplenomegaly, bicytopenia, and the presence of activated macrophages in hematopoietic sites; these constitute the classical HLH criteria.^2^ Among the 2 hemophagocytic syndrome subsets, primary/genetic or secondary/reactive, the latter is the most frequently seen in adults. This condition may be triggered by various underlying conditions such as infection (especially by Epstein-Barr virus (EBV), cytomegalovirus (CMV), or human immunodeficiency virus (HIV)), lymphoid malignancy (B- or T-cell lymphoma), connective tissue diseases, or some drugs.^3^

HLH criteria were established by the Histiocyte Society in 2004.^2^ More recently, Fardet et al^4^ proposed an HScore, a diagnostic tool based on 9 criteria: clinical: known underlying immunosuppression, high temperature, organomegaly; biological: cytopenia, high levels of triglyceride, ferritin, and serum glutamic oxaloacetic transaminase (SGOT), and low level of fibrinogen; and cytological: hemophagocytosis features on bone marrow aspirates. In this study, expert agreement was considered as the gold standard for the diagnosis of HLH. This HScore was the first validated in adult reactive HLH but not yet in familial lymphohistiocytosis.

Because of HLH severity, intensivists are increasingly asked to take care of HLH patients. Indeed, in the absence of specific treatment, the expected outcomes of HLH are multiple organ failure and, ultimately, death;^5,6^ in a recent literature review, Ramos-Casals et al^3^ reported a 41% mortality rate in 1109 patients.

Whereas several studies have investigated the prognostic factors of HLH, little is known about the prognosis of critically ill HLH patients.^7–9^ except for a high hospital mortality rate (52% in 56 patients).^8^ In particular, the HLH mortality-associated risk factors in ICU are still poorly known due to the small sizes of the available series.

The present study describes a relatively large retrospective cohort of HLH patients admitted to several polyvalent ICUs of a group of university hospitals and attempts to identify the early mortality risk factors in HLH.

## METHODS

### Inclusion Criteria

The study reviewed retrospectively the results of all bone marrow aspirations performed in hematology laboratories between January 2002 and July 2014 on the requests of ICUs of 3 university hospitals (Hospices Civils de Lyon, France). All bone marrow aspiration requests for suspicion of HLH and all bone marrow aspirations that showed hemophagocytosis were further examined. All data from patients diagnosed with HLH or whose bone marrow aspirations displayed hemophagocytosis were kept for further analyses. In parallel, the study searched for all patients diagnosed with code D76.1 (hemophagocytic lymphohistiocytosis), D76.2 (hemophagocytic syndrome, infection-associated), or D76.3 (other histiocytosis syndromes) of the International Classification of Diseases, 10th revision (ICD-10).

The study examined retrospectively the medical records of all patients admitted to an ICU 3 months before to 3 months after the date of bone marrow aspiration. As sepsis may mimic some clinical and biological features of HLH,^10,11^ patients with bacteremia (as evidenced by positive blood cultures) within the same week of a bone marrow aspiration were deliberately excluded.

According to the current French legislation (Loi Huriet-Sérusclat 88-1138, 20 December 1988 and its subsequent amendments (http://www.chu-toulouse.fr/IMG/pdf/loihuriet.pdf), an observational study that does not change the routine management of patients does not need to be declared or submitted to the opinion of a research ethics board.

### Positive HLH Diagnosis

All HLH diagnoses were retrospectively checked versus the HScore obtained within 24 hours before or after bone marrow aspiration.^4^ The criteria used for the HScore were: a known underlying immunosuppression (HIV infection or the use of long-term immunosuppressive therapy); the presence of organomegaly, mainly hepatomegaly or splenomegaly; the number of lineages with cytopenia; the maximum ferritin value; the maximum triglyceride value; the minimum fibrinogen value; the maximum SGOT value; and the evidence of hemophagocytosis on bone marrow aspiration. Full details on the criteria for calculating the HScore are given in Table 1.

**TABLE 1:**
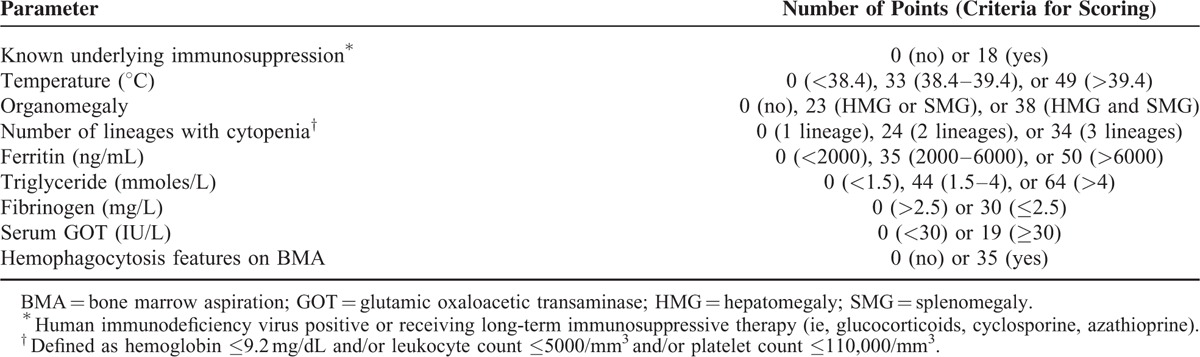
The HScore

The diagnosis of HLH was confirmed in patients with HScore ≥169. This value was considered as the best cut-off value; it corresponded to 93% sensitivity, 86% specificity, and 90% correct classification rate.^4^

### Data Collection

Several clinical and biological data were collected on a standardized form. These data included the nature of the underlying disease, the therapeutic interventions, the variables required for the calculation of the HScore, the reasons for ICU admission, and the organ supportive care provided.

The severity of organ failure was evaluated using the Sequential Organ Failure Assessment (SOFA) score at ICU admission. The occurrence of opportunistic infections, such as invasive aspergillosis, as defined by the EORTC/MSG Consensus Group,^12^ was also checked.

The study considered also some precipitating factors such as the presence of neoplasia, autoimmune disease, bacterial or viral infection, drug reaction (or rash) with eosinophilia and systemic symptoms (DRESS), or an unknown origin for HLH.

Regarding treatment, the study considered the delay to corticosteroid or etoposide administration defined as the time elapsed since HLH diagnosis. Whenever a treatment was administered before HLH diagnosis, this delay was considered zero.

Hospital mortality and mortality within 28 days after ICU admission were specifically examined.

### Statistical Analysis

The study continuous variables were summarized as medians with interquartile ranges and the categorical variables as frequencies and percentages.

The risk-factor analyses used the variables classically considered prognostic factors.^3^ These variables included: the presence of lymphoma-related HLH or HLH of unknown origin (as single factor), thrombocytopenia at diagnosis (<50,000 platelets/microliter), C-reactive protein level >50 mg/L, leukopenia (<500 leukocytes/microliter), renal replacement therapy as categorical variables; and age, anemia, hyperferritinemia, total plasma bilirubin, fibrinogen, and SOFA score at ICU admission as continuous variables. The cut-off values of the categorical variables were taken from the current literature.

The characteristics of the survivors and nonsurvivors (at hospital discharge or within 28 days after ICU admission) were compared in univariate analyses. The Mann–Whitney *U* nonparametric test (test for comparison of medians) was used for the continuous variables and the *χ*^2^ test or Fisher test (as appropriate) for the categorical variables. All variables associated with a *P* value <0.10 were included in a subsequent multivariate analysis using a binary logistic regression. Variable selection used then a backward stepwise selection method. In this multivariate analysis, a *P* value <0.05 was considered for statistical significance. The dataset had very few missing values that could not significantly alter the results of the analyses. Thus, no specific imputation method was used for missing-data imputation.

All the statistical analyses used R Software (version 3.0.2, 2013, R Development Core Team. R: A Language Environment for Statistical Computing. Vienna, Austria. ISBN 3-900051-07-0. URL: http://www.R-project.org 2013).

## RESULTS

### Patient Inclusion

After discarding duplicate records, 115 patients admitted to an ICU were retrieved by the double approach (hematology lab findings and ICD-10 codes). Forty-four cases were discarded because of sepsis (n = 17) and HScore <169 (n = 27).

Finally, 71 patients (50 men and 21 women; mean (min–max) age: 56.2 (24–83) years) with confirmed HLH diagnosis (HScore ≥169) were kept for the statistical analyses. Fifty-one patients (72%) were admitted to an ICU during the last 5 years of the inclusion period.

### Participant Characteristics

The main clinical and laboratory characteristics of confirmed HLH cases (HScore ≥169) and unconfirmed HLH cases (HScore <169) at baseline are shown in Table 2. The HLH criteria (Histiocyte Society, 2004) were met in 32 out of 71 confirmed cases (45%) and 1 out of 27 unconfirmed cases (3.7%) (*P* < 0.001).

**TABLE 2:**
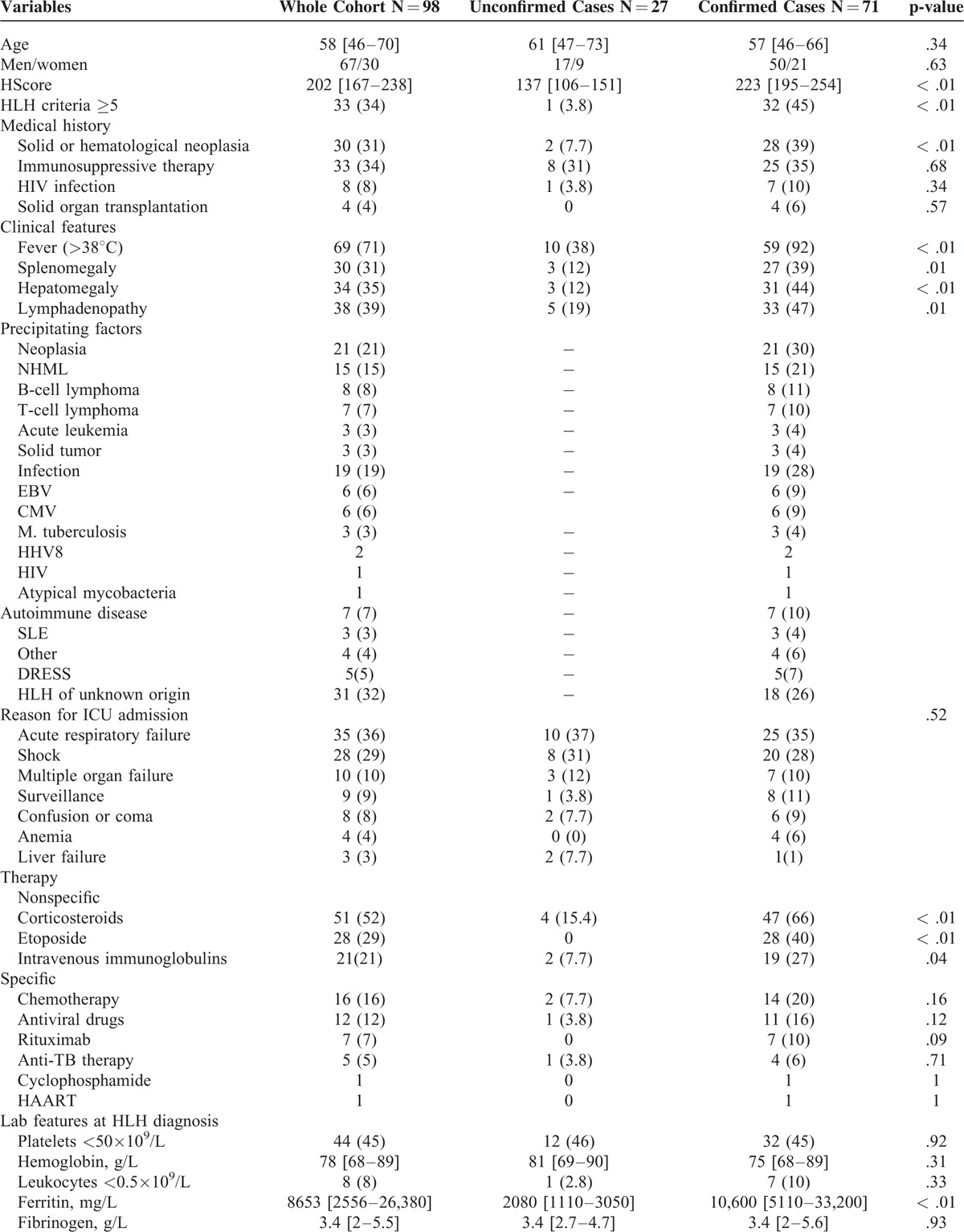
Characteristics of the Whole Cohort, the Confirmed, and the Unconfirmed HLH Cases

**TABLE 2 (Continued):**
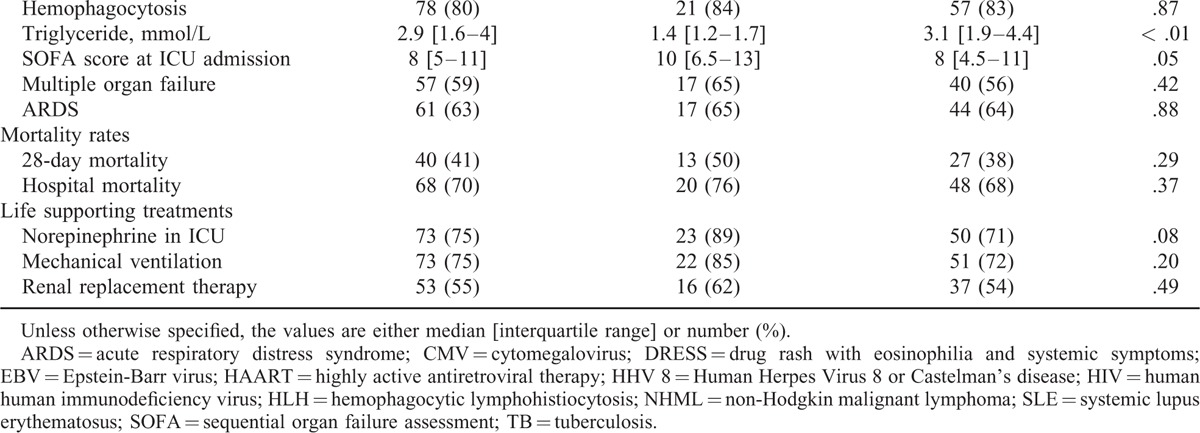
Characteristics of the Whole Cohort, the Confirmed, and the Unconfirmed HLH Cases

The main reasons for ICU admission were acute respiratory failure (36%) and shock (29%); they did not differ significantly between confirmed and unconfirmed cases. The SOFA score at ICU admission was higher in the unconfirmed than in the confirmed cases although not significantly (median: 10 [6.5–13] vs. 8 [4.5–11], *P* = 0.05). The rates of multiple organ failure and acute respiratory distress syndrome (ARDS) did not differ significantly between confirmed and unconfirmed cases.

Corticosteroids and etoposide were much more frequently used in confirmed than in unconfirmed cases (in 47 (66%) vs. 5 (19%) patients, *P* < 0.001; and in 28 (40%) vs. 0, *P* = 0.001; respectively).

Among the confirmed cases, the main precipitating factors were neoplasia (n = 21, 30%) and infection (n = 20, 28%). In 18 cases (26%), no underlying disorder was identified. Among neoplasia cases, non-Hodgkin lymphoma was predominant (n = 16, 23%). The most frequent infections were due to EBV (n = 7, 10%) and CMV (n = 8, 11%).

The course of the disease was complicated by invasive aspergillosis diagnosed according to consensual criteria in 18 confirmed cases (25%).^12^ In these cases, HLH was related to infection (n = 8), solid tumor (n = 4), and autoimmune disease (n = 1); no etiology was found in the 5 remaining cases.

The confirmed and unconfirmed cases showed no significant difference regarding 28-day mortality (n = 27, 38% vs. n = 13, 50%, *P* = 0.29) and hospital mortality (n = 48, 68% versus n = 20, 76%, *P* = 0.37).

### Univariate Analysis Results

Table 3  displays the participant's clinical and biological features at diagnosis or ICU admission according to the outcome. The diagnosis of HLH was made before ICU admission in 10 cases (23%) among the 28-day survivors and 10 cases (37%) among the nonsurvivors.

**TABLE 3:**
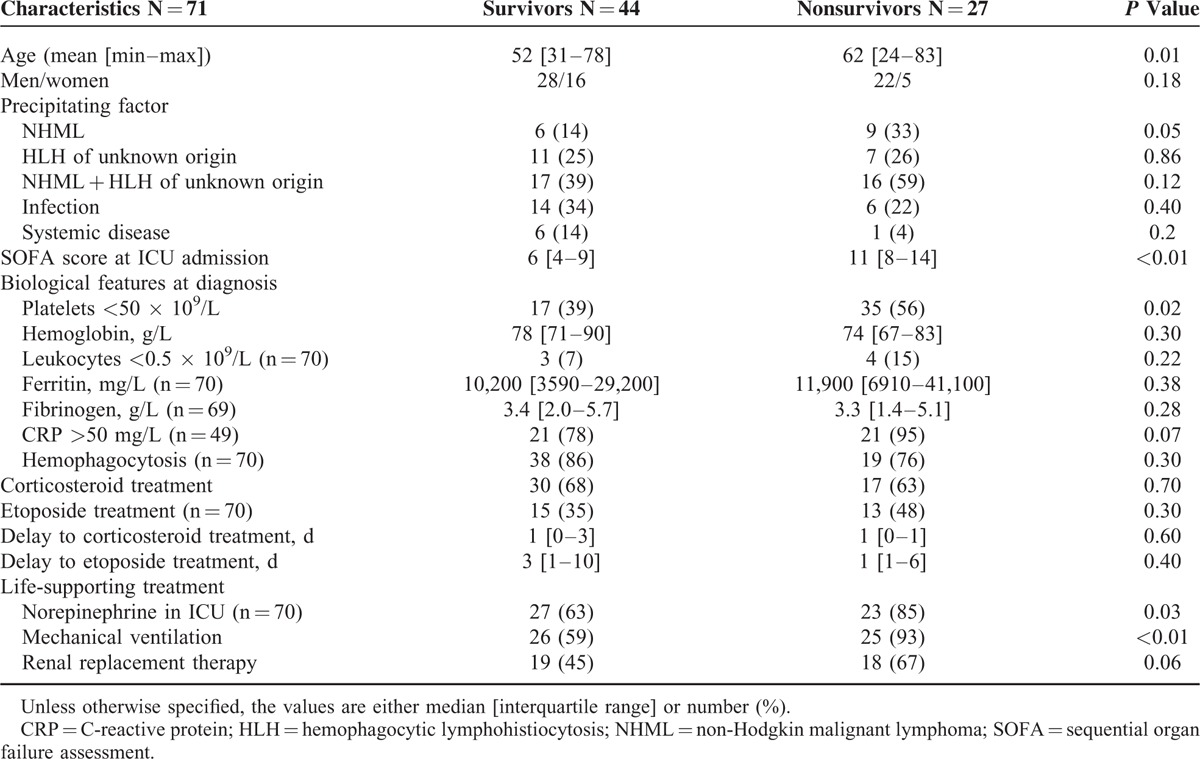
Comparison Between Survivors and Nonsurvivors Within 28 Days After ICU Admission. Univariate Analyses

In the univariate analyses, age (*P* = 0.01) and the SOFA score at ICU admission (*P* < 0.001) were significantly associated with the 28-day mortality whereas thrombocytopenia at diagnosis (*P* = 0.01), the SOFA score at ICU admission (*P* < 0.001), the need for renal replacement therapy (*P* < 0.01), and the presence of lymphoma-related HLH or HLH of unknown origin (*P* < 0.01) were significantly associated with hospital mortality.

Twenty-eight days after ICU admission, invasive aspergillosis had occurred in 11 out of 44 survivors (25%) and 7 out of 27 nonsurvivors (26%) (*P* = 0.93).

### Multivariate Analysis Results

The backward stepwise regression technique identified the advance in age and the SOFA score at ICU admission as the variables that would significantly affect the 28-day mortality. The respective odds ratios [95% confidence intervals] and *P* values were 1.03 [1.00–1.07], *P* = 0.04 and 1.18 [1.03–1.30], *P* < 0.01(Table 4).

**TABLE 4:**
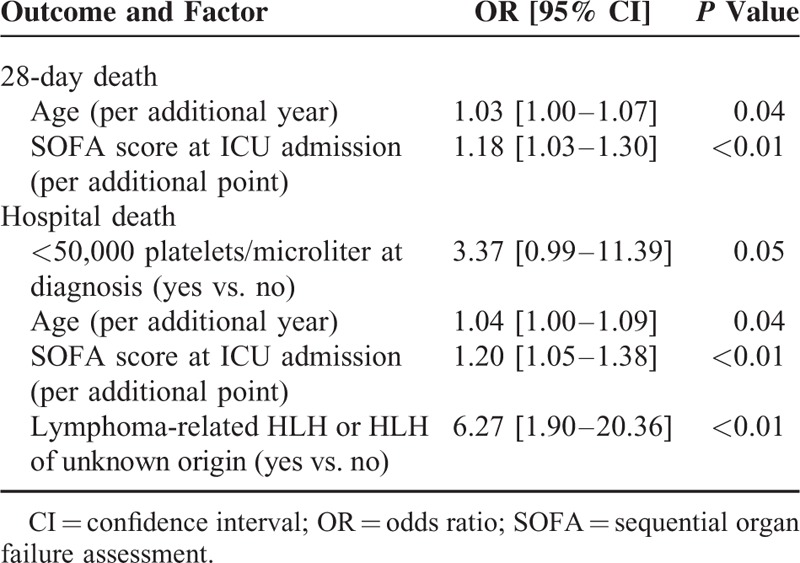
Risk Factors for 28-Day or Hospital Mortality. Multivariate Analysis

The same technique identified the advance in age, the SOFA score at ICU admission, and the presence of lymphoma-related HLH or HLH of unknown origin as the variables that would significantly affect hospital mortality. The respective odds ratios [95% confidence intervals] and *P* values were 1.04 [1.00–1.09], *P* = 0.04, 1.20 [1.05–1.38], *P* < 0.01, and 6.27 [1.90–20.36], *P* < 0.01. Thrombocytopenia at diagnosis (<50,000 platelets/microliter) was almost associated with higher hospital mortality (OR = 3.37 [0.99–11.39], *P* = 0.05 which is of borderline significance).

### Therapeutic Aspects

Corticosteroids were administered to 47 patients, etoposide to 28 patients, and intravenous immunoglobulins to 19 patients.

The mean (min–max) delay between HLH diagnosis and etoposide administration was 6 (0–32) days. The rates of etoposide use and the median delay to its administration were not significantly different between 28-day survivors and nonsurvivors. Similarly, the delay to corticosteroid administration was not significantly different between 28-day survivors and nonsurvivors.

Twenty-eight patients received steroids/etoposide combination. One half of these patients had lymphoma-triggered HLH. The others had HLH of unknown origin (n = 5) treated like lymphoma, infection-associated HLH (n = 7), or autoimmune-disease-associated HLH (n = 2).

Finally, 14 patients received emergency chemotherapy, 12 antiviral treatments, and 7 rituximab.

## DISCUSSION

The present study presents clinical data, biological data, and factors associated with 28-day and hospital mortalities in HLH adult patients who required ICU admission. To our knowledge, it reports on the largest group described so far. Previously, Buyse et al^8^ have reported on 56 HLH patients (as per the HLH-2004 criteria) admitted to the ICU of a highly specialized hematology and immunology hospital.^8^

Here, the diagnosis of HLH relied on the HScore that was recently designed and validated to assess individual risks of HLH. In a previous study, this score and the cut-off value of 169 has ensured rather high values of sensitivity, specificity, and classification accuracy.^4^ The HScore was preferred to the previous HLH-2004 criteria because the latter were suffering from substantial limitations. First, these criteria were established in a pediatric population to diagnose the rather hereditary primary form of HLH and were thus inadequate in adults or in the reactive form of the disease. Second, the weight of each criterion was unknown and the cut-off values were merely empirical. Third, some criteria (eg, NK cell activity, soluble interleukin-2 receptor level) are generally unavailable in daily practice and may be of little interest in diagnosing reactive HLH.

Though the HScore has never been properly validated in ICU patients, the confirmed cases had clinical and biological characteristics that made HLH highly probable; fever, splenomegaly, hepatomegaly, and peripheral lymphadenopathy were present in 92%, 39%, 44%, and 47% of the cases. These proportions are close to the ones reported in the literature.^3,13–15^ In contrast, the unconfirmed cases, defined by an HScore <169, had lower proportions of fever (38%), splenomegaly (12%), or hepatomegaly (12%). Besides, the HLH criteria were met in 45% of the confirmed cases versus 3.8% of the unconfirmed cases.

In the present study, hematologic malignancies, especially non-Hodgkin lymphomas, were by far the main HLH-associated conditions but their proportions were lower than in previous case-series.^3^ Besides, the proportion of patients with known cause of immunosuppression at the time of HLH diagnosis (45%) was smaller than the one reported by Buyse et al (ie, 68%).^8^ On the contrary, the present group included a nonnegligible proportion of patients with HLH of unknown origin (26%), which is close to the proportion recently reported in 103 Chinese adult patients (23.3%) but much higher than the 3.7% and 8% proportions reported respectively by Ramos-Casals et al^3^ and Fardet et al.^15^ In everyday practice, idiopathic HLH cases are often considered nondiagnosed T lymphomas in which last-chance chemotherapy is sometimes attempted. This observation led us to put lymphoma-related and idiopathic HLH cases into the same subgroup before performing the statistical analyses.

Here, the main reasons for ICU admission were acute respiratory failure (35%) and shock (29%); these proportions are close to those reported by Buyse et al.^8^ The incidence of invasive aspergillosis was also high (n = 18, 25%). Invasive aspergillosis was mainly seen in infection-related HLH (n = 8) rather than in HLH of unknown origin (n = 5) or in lymphoma-related HLH (n = 4). The association between invasive aspergillosis and HLH was described in only a few case reports^16,17^ in which it was associated with hematological malignancies.

In previous studies, the overall reactive-HLH-associated mortality ranged between 22% and 60%.^15^ Here, the choice was made to study early mortality, which is more likely linked to the severity of HLH rather than to the underlying diseases or treatment complications. However, in most cases, no single specific cause of death could be identified. Here, the mortality rate was not significantly different between confirmed and unconfirmed cases; however, there was a trend toward a higher mortality rate in unconfirmed cases (which may be explained by higher SOFA scores).

Data about CNS involvement were available only at ICU admission. After admission, most of the patients needed sedation due to severe hemodynamic or respiratory failure making difficult any neurological assessment. Neurological failure was the reason for ICU admission in 6 of the 71 cases: 3 cases of convulsive status epilepticus and 3 cases of unexplained coma. In the univariate analyses, these 6 conditions were not significantly associated with poorer outcome.

Patients with lymphoma-related or HLH of unknown origin tended to have shorter short-term survivals than patients with infection-associated HLH. As mentioned above, the favorable prognosis of Castleman-disease-related HLH, as described by Buyse et al^8^ and Arca et al,^18^ may be partly explained by a systematic and early use of etoposide, possibly associated with rituximab.^18^

Here, contrary to previous reports, none of hyperferritinemia, hyperbilirubinemia, high C-reactive protein level, or hemophagocytosis features on bone marrow aspirations was significantly associated with higher mortality.^3^ The advance in age, the SOFA score at ICU admission, lymphoma-related HLH, or HLH of unknown origin were also associated with higher risks of hospital mortality. The already reported poor prognosis of lymphoid-malignancy-associated HLH^15^ may be partly explained by the proportion of T-cell lymphomas that have poor prognoses per se due to high refractoriness to initial chemotherapies.^19^ Thrombocytopenia <50,000 platelets/microliter was almost associated with increased hospital mortality. In previous studies, this factor was often found associated with poor prognosis.^8,15,20–22^

Etoposide therapy was used in 41% of the present HLH group versus 80% in Buyse's report. Here, the recourse to etoposide was not found associated with a decrease of mortality rates; however, Buyse et al^8^ and, more recently, Arca et al^18^ found a trend toward longer survival in patients given etoposide. This difference may be explained by the high degree of specialization of the hospitals where the latter studies were carried out; in these hospitals, the physicians seem to be quite familiar with HLH management.

The present study has several limitations. One is related to the patient selection and the use of the HScore instead of HLH-2004 criteria. This prevents relevant comparisons with previous results; however, in our setting, the comparison between confirmed and unconfirmed cases supported the accuracy of the HScore in diagnosing HLH. This new score will probably help including more patients in future studies. Another limitation is the low number of participants. Actually, though this group is among the largest ICU-admitted HLH patients known so far, the numbers of patients in the survivor and nonsurvivor groups were low and did not allow accurate statistical estimations. Nevertheless, 1 asset of the present study is its multicentric design (7 ICUs in 3 university hospitals with no hematological specialization); this provides an unbiased idea of the way HLH is currently managed. Furthermore, the majority of the patients were included during the 5 last years of the inclusion period; this reduced the possible bias related to the technological evolution and the variations in the clinical approach to critical patients that play important roles as determinants of mortality. Relatively poor usage of chemotherapy in conventional ICUs should lead to the transfer of these patients to specialized centers more familiar with the management of acute hematological malignancies.

In conclusion, the present results that stem from a broad spectrum of clinical conditions were able to identify reliable prognostic factors of early death that may help a quick identification of patients requiring ICU admission. The 28-day mortality was linked to classical ICU prognostic factors, whereas hospital mortality was, in addition, linked to the underlying disease. These results suggest that organ failure overtops the classical prognostic factors identified in adult patients with HLH. This failure may be prevented by timely HLH diagnosis, use of specific cytotoxic therapies, and the control of the underlying disease with, as outcome, a reduction of early HLH-related mortality.

## References

[1] Filipovich AH Hemophagocytic lymphohistiocytosis (HLH) and related disorders. *Hematology* 2009; 2009:127–131. 20008190 10.1182/asheducation-2009.1.127

[2] Henter JI Horne A Arico M HLH-2004: diagnostic and therapeutic guidelines for hemophagocytic lymphohistiocytosis. *Pediatr Blood Cancer* 2007; 48:124–131. 16937360 10.1002/pbc.21039

[3] Ramos-Casals M Brito-Zeron P Lopez-Guillermo A Adult haemophagocytic syndrome. *Lancet* 2014; 383:1503–1516. 24290661 10.1016/S0140-6736(13)61048-X

[4] Fardet L Galicier L Lambotte O Development and validation of a score for the diagnosis of reactive hemophagocytic syndrome (HScore). *Arthritis Rheumatol* 2014; 66:2613–2620. 24782338 10.1002/art.38690

[5] Creput C Galicier L Buyse S Understanding organ dysfunction in hemophagocytic lymphohistiocytosis. *Intensive Care Med* 2008; 34:1177–1187. 18427781 10.1007/s00134-008-1111-y

[6] Lane S Andrist C Nagarajan A Hemophagocytic lymphohistiocytosis (HLH) in a 25-year-old presenting with multisystem organ failure. *West Virginia Med J* 2013; 109:22–23. 24371860

[7] Imashuku S Hlbi S Todo S Hemophagocytic lymphohistiocytosis in infancy and childhood. *J Pediatr* 1997; 130:352–357. 9063408 10.1016/s0022-3476(97)70195-1

[8] Buyse S Teixeira L Galicier L Critical care management of patients with hemophagocytic lymphohistiocytosis. *Intensive Care Med* 2010; 36:1695–1702. 20532477 10.1007/s00134-010-1936-z

[9] Trottestam H Horne A Arico M Chemoimmunotherapy for hemophagocytic lymphohistiocytosis: long-term results of the HLH-94 treatment protocol. *Blood* 2011; 118:4577–4584. 21900192 10.1182/blood-2011-06-356261 PMC3208276

[10] Usmani GN Woda BA Newburger PE Advances in understanding the pathogenesis of HLH. *Br J Haematol* 2013; 161:609–622. 23577835 10.1111/bjh.12293

[11] Castillo L Carcillo J Secondary hemophagocytic lymphohistiocytosis and severe sepsis/systemic inflammatory response syndrome/multiorgan dysfunction syndrome/macrophage activation syndrome share common intermediate phenotypes on a spectrum of inflammation. *Pediatr Crit Care Med* 2009; 10:387–392. 19325510 10.1097/PCC.0b013e3181a1ae08

[12] De Pauw B Walsh TJ Donnelly JP Revised definitions of invasive fungal disease from the European Organization for Research and Treatment of Cancer/Invasive Fungal Infections Cooperative Group and the National Institute of Allergy and Infectious Diseases Mycoses Study Group (EORTC/MSG) Consensus Group. *Clin Infect Dis* 2008; 46:1813–1821. 18462102 10.1086/588660 PMC2671227

[13] Riviere S Galicier L Coppo P Reactive hemophagocytic syndrome in adults: a multicenter retrospective analysis of 162 patients. *Am J Med* 2014; 127:1118–1125. 24835040 10.1016/j.amjmed.2014.04.034

[14] Otrock ZK Eby CS Clinical characteristics, prognostic factors, and outcomes of adult patients with hemophagocytic lymphohistiocytosis. *Am J Hematol* 2015; 90:220–224. 25469675 10.1002/ajh.23911

[15] Li J Wang Q Zheng W Hemophagocytic lymphohistiocytosis: clinical analysis of 103 adult patients. *Medicine* 2014; 93:100–105. 24646466 10.1097/MD.0000000000000022 PMC4616310

[16] Halasa NB Whitlock JA McCurley TL Fatal hemophagocytic lymphohistiocytosis associated with Epstein-Barr virus infection in a patient with a novel mutation in the signaling lymphocytic activation molecule-associated protein. *Clin Infect Dis* 2003; 37:e136–e141. 14583885 10.1086/379126

[17] Larbcharoensub N Aroonroch R Kanoksil W Infection-associated hemophagocytic syndrome among patients with dengue shock syndrome and invasive aspergillosis: a case series and review of the literature. *Southeast Asian J Trop Med Public Health* 2011; 42:1106–1112. 22299436

[18] Arca M Fardet L Galicier L Prognostic factors of early death in a cohort of 162 adult haemophagocytic syndrome: impact of triggering disease and early treatment with etoposide. *Br J Haematol* 2014; 168:63–68. 25157895 10.1111/bjh.13102

[19] Mak V Hamm J Chhanabhai M Survival of patients with peripheral T-cell lymphoma after first relapse or progression: spectrum of disease and rare long-term survivors. *J Clin Oncol* 2013; 31:1970–1976. 23610113 10.1200/JCO.2012.44.7524

[20] Tiab M Mechinaud F Hamidou M Hemophagocytic syndromes. A series of 23 cases. *Ann Med Interne* 1996; 147:138–144. 8796089

[21] Tseng YT Sheng WH Lin BH Causes, clinical symptoms, and outcomes of infectious diseases associated with hemophagocytic lymphohistiocytosis in Taiwanese adults. *J Microbiol Immunol Infect* 2011; 44:191–197. 21524613 10.1016/j.jmii.2011.01.027

[22] Dhote R Simon J Papo T Reactive hemophagocytic syndrome in adult systemic disease: report of twenty-six cases and literature review. *Arthritis Rheum* 2003; 49:633–639. 14558048 10.1002/art.11368

